# Public Attitude Toward Elder Family Financial Exploitation: Analysis of Social Media Data

**DOI:** 10.1093/geroni/igaf122.2255

**Published:** 2025-12-31

**Authors:** Tina Kilaberia, Weicheng Zeng, Ruopeng An

**Affiliations:** New York University, New York, New York, United States; New York University, New York, New York, United States; New York University, New York, New York, United States

## Abstract

Underreporting of privacy-laden family financial exploitation of older adults and Federal underinvestment in response to elder mistreatment can adversely influence older people’s help-seeking, health, and mental health outcomes. This study compared public attitudes toward family financial exploitation by focusing on one victim (21 YouTube videos), one perpetrator (8), and a case of family financial abuse generally (19), totaling 3,796 comments included in the analysis. The victims and perpetrators in the videos were not related. Three levels of analysis were conducted: sentiment and emotion analyses using advanced natural language processing (NLP) methods and a human-coded subsample. Sentiment analysis showed that among the three cases, a greater share of negative (about 40%) and positive (nearly 60%) comments was attributed to the perpetrator and victim, respectively. Emotion analysis found no shared emotions between the victim and perpetrator cases. Disapproval, surprise, disgust, and confusion were expressed solely concerning the perpetrator, whereas anger and fear were expressed solely concerning the victim. Caring, love, and gratitude were shared emotions across the victim and the general cases. Sadness, admiration, curiosity, annoyance, and approval were five emotions shared across all three cases. Human-coded sub-sample analysis showed expressions of sarcasm, and microaggressions directed at the perpetrator’s race and gender. Although NLP methods did not capture victim-perpetrator background-related attitudes in relation to family financial abuse, they delineated relatively clear-cut attitudes toward victim and perpetrator status, reflecting a public concern toward elder family financial exploitation. Public attitudes may be harnessed for stakeholder engagement to increase reporting and investment in elder mistreatment.

